# Optimization of Solid Lipid Microcapsule Matrix for Enhanced Release and Bioavailability of L-Lysine in Swine

**DOI:** 10.3390/ani15121806

**Published:** 2025-06-19

**Authors:** Costanza Bonnici, Maria Federica Marchesi, Martina Felici, Federico Ghiselli, Roberta Majer, Benedetta Tugnoli, Guglielmo Gallina, Andrea Piva, Ester Grilli

**Affiliations:** 1 Dipartimento di Scienze Mediche Veterinarie (DIMEVET), Università di Bologna, via Tolara di Sopra 50, Ozzano dell’Emilia, 40064 Bologna, Italy; maria.marchesi3@unibo.it (M.F.M.); ester.grilli@unibo.it (E.G.); 2 Vetagro S.p.A., via Porro 2, 42124 Reggio Emilia, Italy; martina.felici@vetagro.com (M.F.); federico.ghiselli@vetagro.com (F.G.); roberta.majer@vetagro.com (R.M.); benedetta.tugnoli@vetagro.com (B.T.); ap@vetagro.com (A.P.); 3 Vetspin Srl, via Toscanini 9, Villanova di Castenaso, 40055 Bologna, Italy; guglielmo.gallina@vetspin.com; 4 Vetagro Inc., 936 SW 1st Ave, Suite 878, Miami, FL 33130, USA

**Keywords:** lysine, microcapsules, feed additives, amino acid supplementation, bioavailability, functional properties, nutrient requirements, growth

## Abstract

L-lysine (L-Lys) is the first-limiting amino acid in swine nutrition, but free-form supplements exhibit poor intestinal absorption, reducing their bioavailability. This study aimed to enhance the gastric retention, controlled intestinal release, and systemic availability of L-Lys by optimizing solid lipid microcapsules (SLMs). SLMs were formulated using hydrogenated triglycerides (C16:0 or C18:1), free fatty acids, and varying emulsifier concentrations. Gastric retention and intestinal release were evaluated in vitro under simulated gastrointestinal conditions (a pepsin buffer at pH 5.0 for 2 h, followed by a pancreatin buffer at pH 6.5 for up to 8 h at 39 °C). SLMs with hydrogenated triglycerides showed significantly higher gastric retention (94–95%) than those with free fatty acids (48%). Specifically, C16:0 triglyceride-based SLMs achieved 74% intestinal release, which was enhanced to 90% with 1% emulsifier. This refined formulation was subsequently evaluated in vivo using weaned pigs (three groups; n = 4) fed a basal cornmeal diet. The treatments included a single oral administration of saline solution (placebo), free L-Lys (0.17 g/kg BW), or L-Lys SLMs (0.38 g/kg BW, equally providing L-Lys at 0.17 g/kg BW). The SLMs delayed the L-Lys plasma peak (T. max. 3–4 h vs. 1 h) and significantly increased the total L-Lys amount in the plasma over 24 h, demonstrating the enhanced relative bioavailability of encapsulated L-Lys.

## Simple Summary

This study explored the use of solid lipid microcapsules (SLMs) to enhance the effectiveness of L-lysine (L-Lys) supplementation in swine nutrition. The SLMs were formulated with different lipid matrices and varying levels of an emulsifier to improve the L-Lys SLMs’ retention in the stomach and control their release in the intestine. Results from both in vitro and in vivo experiments showed that optimizing the lipid composition and emulsifier concentration significantly improved L-Lys delivery during the digestion phase and delayed its availability in plasma. Encapsulated L-Lys showed markedly higher relative bioavailability than the free form, highlighting the importance of SLM matrix optimization to improve L-Lys supplementation in swine.

## 1. Introduction

L-lysine (L-Lys) is the first-limiting amino acid (AA) in swine nutrition, meaning that its dietary availability directly influences protein synthesis and muscle growth [1]. For this reason, L-Lys deficiency can impair growth performance, reduce feed efficiency, and increase physiological stress, making its supplementation essential [1].

Despite its critical role, supplementation with crystalline L-Lys presents several limitations, including possible degradation in the acidic gastric environment, potentially reducing its availability for absorption in the small intestine, the primary site for AA uptake [2]. Furthermore, at a high concentration of supplementation, the free L-Lys influx into the intestine may saturate intestinal AA transporters, potentially impairing its absorption efficiency and, consequently, increasing L-Lys excretion [3,4].

This inefficiency affects protein synthesis and contributes to environmental nitrogen pollution via increased fecal output [5]. In addition, in monogastric animals such as broilers and pigs, free AAs are absorbed rapidly, leading to imbalances and a reduced utilization efficiency due to oxidation [5,6].

To overcome these challenges, microencapsulation technologies have emerged as promising strategies to enhance the stability, absorption, and utilization of AAs [7]. Among these, lipid-based encapsulation offers several key advantages. For instance, solid lipid microcapsules (SLMs) are specifically designed to shield bioactive compounds from harsh gastric conditions, leveraging the inherent resistance of lipids to stomach digestion. Conversely, lipases facilitate lipid breakdown within the intestinal tract, enabling the controlled release of the bioactive compounds. This entire process leads to improved systemic bioavailability [7]. However, SLMs’ performance is heavily influenced by the physicochemical properties of their lipid matrix. Specifically, triglycerides’ melting behavior and structural stability are influenced by the length of fatty acid chains (with longer chains elevating melting points) and the degree of saturation (where saturated lipids enhance molecular packing). These factors collectively determine the properties of the matrix and strongly influence its structural integrity and disintegration behavior, leading to targeted nutrient delivery and increasing the absorption efficiency [8].

Saturated long-chain triglycerides (e.g., C16:0) tend to form semi-crystalline matrices with high melting points, enhanced molecular packing, and resistance to acid degradation [6,7]. Their structure enables their controlled disintegration in the intestine, making them a popular choice for microencapsulation [9]. In contrast, matrices based on monounsaturated lipids (e.g., C18:1) feature a double bond that requires greater activation energy to cleave than a single bond [10]. As a result, these lipids may exhibit increased stability against lipase-mediated degradation. Meanwhile, a matrix predominantly composed of saturated free fatty acids creates rigid structures, which may fracture prematurely under gastric stress, reducing their protective efficacy [11,12].

Incorporating emulsifiers could add another layer of control due to their amphiphilic nature, which could decrease interfacial tension, enhance matrix cohesion, improve encapsulation efficiency, and interact with bile salts to promote gradual lipid disintegration [13,14]. Thus, tailoring the lipid composition with compatible emulsifiers is critical to balancing gastric retention with sustained intestinal delivery.

Therefore, this study aimed to characterize the physicochemical properties of the SLM matrix, focusing on compositions based on hydrogenated vegetable oils rich in either C16:0 triglycerides, C18:1 triglycerides, or free fatty acids. These components are likely to influence the behavior of SLMs, particularly their structural integrity, gastric retention, and release profiles. Furthermore, this study examined how two distinct emulsifier concentrations modulate intestinal lipid disintegration. The final part of this research investigated the in vivo relative bioavailability of L-Lys for the most promising formulation identified in vitro, evaluating its performance under in vivo conditions. In vitro simulations of gastrointestinal conditions were used to screen candidate matrices. The most promising formulation was subsequently tested in vivo via post-prandial plasma kinetics and was compared with free L-Lys supplementation in swine.

## 2. Materials and Methods

### 2.1. SLMs’ Development and Characterization

The SLMs used in this study were manufactured by Vetagro S.p.A. (Reggio Emilia, Italy) using a proprietary spray-chilling microencapsulation process.

Briefly, after heating the lipid matrix, L-Lys HCl was mixed with the molten lipid phase and binders, including precipitated and dried silicic acid (E 551a). The mixture was sprayed into a chilling chamber to induce solidification. The resulting SLMs were sealed and stored at 4 °C. Proximate analysis revealed the SLMs consisted of 95% crude fat and 5% ash (*w*/*w*, dry basis). All SLMs were produced in the same process conditions (nozzle and chamber temperatures, spray rate, and air pressure), which were not disclosed by the manufacturer. Relating the process parameters to the product properties was outside of the scope of this study, which focused on the SLMs’ physicochemical properties in relation to their functions during digestion, as described below. The formulations were designed to incorporate 50% L-Lys HCl (*w*/*w*), yielding microcapsules with a final L-Lys content of 40% (*w*/*w*).

Different prototypes were developed by varying the lipid matrix (C16:0-rich vs. C18:1-rich hydrogenated vegetable oils vs. free fatty acids) and emulsifier levels (0%, 0.5%, or 1% *w*/*w*) to assess the effects of the matrix composition and emulsifier concentration on the SLMs’ performance, as summarized in Table 1. In vitro screening was initially performed to identify the most promising matrix (prototypes 1–3), followed by additional testing of emulsifier-enriched variants of the optimal formulation (prototypes 4 and 5). A natural, plant-derived emulsifier (feed-grade, non-GMO, HLB 3–4) was selected, characterized by an amphiphilic structure with hydrophilic and lipophilic groups.

The encapsulation efficiency (EE) was used to assess the production robustness by considering the expected (theoretical) L-Lys concentration based on the amount added during formulation and the analytical (actual) L-Lys found in the SLMs. The calculation was as follows:

(1) $$Encapsulation\;Efficiency\;\left(\%\right)=\frac{actual\;L\text{-}Lys\;content\;\left(\%\right)}{theoretical\;L\text{-}Lys\;content\;\left(\%\right)}\times 100$$

### 2.2. In Vitro Release in Simulated Gastrointestinal Conditions

The in vitro release profile of L-Lys from the SLMs was evaluated using simulated gastric fluid (SGF) and intestinal fluid (SIF), adapted from the Infogest 2.0 protocol for humans [15]. To reflect swine gastric conditions, the SGF was prepared with pepsin derived from porcine gastric mucosa (32 mg/mL; activity (U) ≥ 2500 U/mg protein), and its pH was brought to pH 5.0 with 0.1 M NaOH. This pH accounted for the dietary buffering capacity that elevates the gastric pH to 4–6 post-feeding [16]. The SIF was prepared using pancreatin from porcine pancreas 8 × USP (4 mg/mL; 200 U/mg protein) and bile salts (151 mg/mL; 1.1 mmol/g protein), extracted from purified fresh bile. The SIF’s pH was then adjusted to pH 6.5 with 1 M HCl. All chemicals were purchased from Merk (Milan, Italy).

A sample of 0.5 g was weighed into nylon bags (with a 50 µm pore size) for each SLM prototype, ensuring its exposure to the digestive media while preventing particle loss. The samples were incubated at a 1:10 (g/mL) ratio, with four replicates per condition.

The SGF phase was carried out at 39 °C for 2 h with continuous agitation to mimic gastric peristalsis. The bags were then transferred to SIF and incubated at 39 °C for either 4 h or 6 h to simulate the total gastrointestinal digestion times of 6 h (2 h SGF + 4 h SIF) and 8 h (2 h SGF + 6 h SIF), respectively. Similarly, continuous agitation was performed during the SIF phase to mimic intestinal peristalsis. Post-incubation, the bags were rinsed and dried overnight at 40 °C for subsequent nitrogen analysis.

### 2.3. L-Lysine Analysis and Quantification

The L-Lys content in each sample was quantified via the Kjeldahl method [17], following the AOAC guidelines. The post-digestion dried samples (~0.5 g) were analyzed in quadruplicate. The total nitrogen (N) was converted to an L-Lys percentage using the following conversion:

(2) $$L\text{-}Lys\;(\%)=\frac{N\left(g\right)*6.52}{sample\;weight\;(g)}$$

where 6.52 is the molecular weight ratio between L-Lys HCl and NH₃.

The L-Lys retention (%) was calculated at each time point (TP) relative to the initial L-Lys content (0 h) in the sample. The gastric retention (%) was determined after 2 h in the SGF, and the total release (%) was calculated by subtracting the retention (%) from 100%. The amount of L-Lys released from SLMs during the intestinal phase indicates its in vitro bioaccessibility, reflecting the proportion of L-Lys that is potentially accessible for uptake and utilization by the body.

The calculations were performed as follows:

(3) $$Retention\;\left(\%\right)=\frac{{\%\;L\text{-}Lys\;Retained}_{TP}}{{\%\;L\text{-}Lys}_{0h}}$$

(4) $$Gastric\;Retention\;\left(\%\right)=\frac{{\%\;L\text{-}Lys\;Retained}_{2h}}{{\%\;L\text{-}Lys}_{0h}}$$

(5) Total Release (%)=100−Retention (%)

Graphs depicting the retention percentages can be found in the Supplementary Materials, Figures S1 and S2.

### 2.4. In Vivo Trial

This in vivo trial was conducted in collaboration with VETSPIN Srl (Villanova di Castenaso, BO, Italy). The animals were managed according to Directive 2010/63/EU regarding the protection of animals used for scientific purposes (enforced by the Italian D.Lgs. n° 26 of 4 March 2014). The procedures described in this protocol were reviewed and approved by Vetspin’s Animal Care and Committee (OPBA), as well as by the Italian Ministry of Health (authorization n°254/2022-PR).

A total of 12 healthy male weaned pigs (*Sus scrofa domesticus*) with an average body weight of 15 ± 2 kg BW (approximately 50 days of age) were randomly assigned to three treatment groups, with 4 animals per group (n = 4). The animals were housed individually in 1.5 m^2^ pens under controlled environmental conditions (22 °C; 12 h light/dark cycle) and monitored daily for their health and behavior. All pigs received a standard basal diet without antibiotics, growth promoters, or additives for 7 days before treatment. To minimize the dietary L-Lys background, the feed was switched to whole corn, a low-Lys cereal, 24 h before treatment and maintained during sampling. The corn diet and water were provided ad libitum.

The trial evaluated three different dietary treatments: (1) a placebo group, receiving an oral dose of saline solution; (2) a treatment group, receiving free L-Lys (99% feed grade) at a dose of 0.17 g/kg BW; and (3) a treatment group receiving microencapsulated L-Lys (SLM prototype 5) at a dose of 0.38 g/kg BW. The higher SLM dosage (0.38 vs. 0.17 g/kg BW) accounted for the fact that L-Lys constituted only a fraction of the total microparticle mass (~50% lipid matrix). Prototype 5 was selected due to its improved gastric retention and intestinal release properties, as demonstrated in the in vitro trial conducted in the first part of this study. Blood samples were collected from the jugular veins of each pig, following a single oral administration of the respective treatments, at predefined time points: 0 (pre-treatment), 0.5, 1, 2, 3, 4, 6, 8, 10, 12, 15, and 24 h post-administration. Following collection, the blood samples were immediately processed via centrifugation at 1000× *g* for 15 min at room temperature. The plasma supernatant was collected and promptly frozen at −80 °C until analysis.

### 2.5. LC–MS/MS Analysis of Plasma L-Lys

The plasma L-Lys concentrations were measured via LC–MS/MS using a Waters™ UPLC^®^ system coupled to a TQ-S Cronos triple quadrupole mass detector (ESI+ mode), purchased from Waters™ (Milan, Italy). The full analytical parameters are listed in the Supplementary Materials, Table S1.

A 100 ppm stock solution of L-Lys was prepared in 0.1 M HCl. Two matrix-matched calibration curves were constructed using spiked bovine plasma (curve 1: 10, 7.5, and 5 ppm; curve 2: 12.5, 6.25, and 3.125 ppm) to correct for potential matrix effects.

The samples were processed via protein precipitation (plasma–acetonitrile ratio was 1:5 *v*/*v*), followed by centrifugation (1500× *g*; 10 min; 20 °C). The supernatants were derivatized using AccQTag reagent (Waters™, Milan, Italy). Briefly, a mixture of 10 µL of the samples/standard, 70 µL of borate buffer, and 20 µL of the derivatizing agent was vortexed (10–15 s), incubated at 55 °C for 10 min, and then diluted with 400 µL of water. The preparation of the borate buffer and derivatizing reagent followed the manufacturer’s instructions [18].

Both undiluted and 1:4-diluted samples were analyzed. The quantification was based on the average of both calibration curves to enhance the analytical robustness.

The plasma L-Lys concentration was quantified using liquid chromatography–tandem mass spectrometry (LC-MS/MS).

### 2.6. Statistical Analysis

All statistical analyses were conducted using GraphPad Prism 10.5 (GraphPad Software, Boston, MA, USA). Outliers were screened using Grubbs’ test (α = 0.05). The data normality was verified with the Shapiro–Wilk test and the variance homogeneity with Levene’s test.

The in vitro data (gastric retention, total retention, and release) were analyzed via two-way ANOVA with Tukey’s post hoc test. The values are expressed as means ± SEM (n = 4). The in vivo plasma L-Lys concentrations were analyzed using two-way repeated-measure ANOVA and mixed-model analysis, with time and treatment as factors, followed by Šídák’s multiple-comparison test. The results are reported as means ± SEM (n = 4). Significance was set at *p* < 0.05 for all the analyses.

## 3. Results

### 3.1. Encapsulation Efficiency

The SLM EE value for each prototype is listed in Table 2. All formulations demonstrated a high EE, ranging from 97.81% to 102.33%, closely aligning with the theoretical target of 100%. The narrow standard deviations (SD ≤ 1.26) across the replicates highlight the reproducibility of the spray-chilling process.

### 3.2. In Vitro Test: Effect of Matrix Composition on Gastric Retention and Intestinal Release

**Figure 1:**
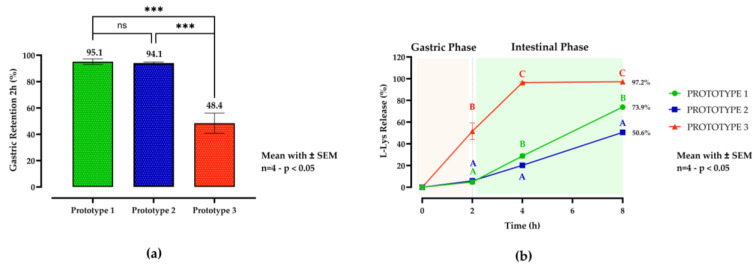
In vitro gastric retention (**a**) and release profiles (**b**) of L-lysine (L-Lys) from solid lipid microcapsule (SLM) prototypes during simulated gastrointestinal digestion. Capsules were subsequently incubated for 2 h in simulated gastric fluid (SGF; pH 5.0) and in simulated intestinal fluid (SIF; pH 6.5) for up to 6 h at 39 °C. Data represent means ± SEM (n = 4). Prototype 1 (green column and line plot with circles) was formulated with hydrogenated vegetable oil rich in saturated triglycerides (C16:0-TGs), prototype 2 (blue column and line plot squares) with hydrogenated oil rich in monounsaturated triglycerides (C18:1-TGs), and prototype 3 (red column and plot with triangles) with free fatty acids (≥95% C18:0). For gastric retention, a one-way ANOVA followed by Tukey’s post hoc test for multiple comparisons was performed (*p* < 0.05). The L-Lys release profiles were analyzed using a two-way ANOVA followed by Tukey’s post hoc analysis. Different letters (A, B, and C) denote statistically significant differences at each time point (*p* < 0.05). *** indicates a highly significant difference (*p* < 0.001), while ns indicates no significant difference. Statistical significance was set at *p* < 0.05.

The detailed results are shown in Figure 1.

A preliminary in vitro experiment evaluated the effect of the matrix composition on the gastric retention and intestinal release of L-Lys. The gastric retention results are presented in the lower left section of Figure 1a. Prototypes 1 and 2 (C16:0- and C18:1-rich triglycerides, respectively) showed excellent gastric retention, with 95.1% and 94.1% retention after 2 h and no statistically significant difference between them (*p* = 0.9854). In contrast, prototype 3 (free fatty acids) exhibited significantly lower gastric retention (48.4%; *p* = 0.0001 vs. prototype 1, and *p* = 0.0002 vs. prototype 2). The release kinetics are shown in the lower right section of Figure 1b. Prototype 3 showed rapid and nearly complete release (96.4%) within 4 h, significantly exceeding both prototypes 1 and 2 at all time points (*p* < 0.0001 at 4 h and 8 h). In contrast, prototypes 1 and 2 demonstrated slower, more controlled release, with prototype 1 consistently releasing the L-Lys at a significantly slower rate than the other prototypes (*p* = 0.0434 vs. prototype 2 at 4 h; *p* < 0.001 vs. prototypes 2 and 3 at 8 h).

The two-way ANOVA results show that time accounted for 60.09% of the total variation (*p* < 0.0001), prototype for 25.85% (*p* < 0.0001), and the time × prototype interaction for 12.65% (*p* < 0.0001), indicating that both factors and their interaction significantly influenced L-Lys release.

### 3.3. Effect of Emulsifier on Gastric Retention and Intestinal Release

**Figure 2:**
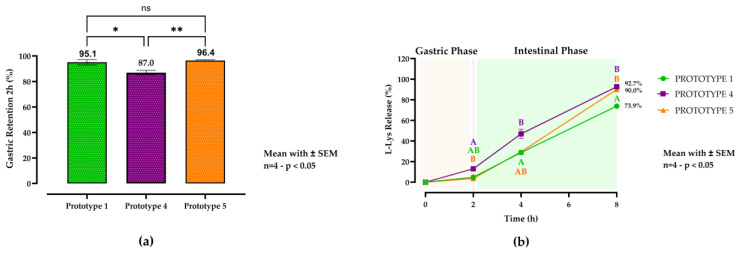
In vitro gastric retention (**a**) and release profiles (**b**) of L-lysine (L-Lys) from solid lipid microcapsule (SLM) prototypes during simulated gastrointestinal digestion. The capsules were subsequently incubated for 2 h in simulated gastric fluid (SGF; pH 5.0) and simulated intestinal fluid (SIF; pH 6.5) for up to 6 h at 39 °C. Data represent means ± SEM (n = 4). Prototype 1 (green column and line plot with circles) contained no emulsifier, prototype 4 (purple column and line plot with squares) included 0.5% emulsifier, and prototype 5 (orange column and line plot with triangles) contained 1% emulsifier. For gastric retention, a one-way ANOVA followed by Tukey’s post hoc test for multiple comparisons was performed (*p* < 0.05). The L-Lys release profiles were analyzed using a two-way ANOVA followed by Tukey’s post hoc analysis. Different letters (A, B) denote statistically significant differences at each time point (*p* < 0.05). ** indicates a very significant difference (*p* < 0.01), * indicates a significant difference (*p* < 0.05), while ns indicates no significant difference. Statistical significance was set at *p* < 0.05.

The detailed results are shown in Figure 2.

In the second in vitro trial, the emulsifier concentration was evaluated using the C16:0-based matrix (prototype 1). The gastric retention results are presented in the lower left section of Figure 2a. Prototype 4 (0.5% emulsifier) exhibited lower gastric retention (87%) than prototypes 1 (95.1%) and 5 (1% emulsifier; 96.4%). The statistical analysis revealed a significant difference in gastric retention between prototypes 1 and 4 (*p* = 0.0167). However, the gastroprotective effects of prototypes 1 and 5 did not significantly differ (*p* = 0.8414). Furthermore, a significant difference was observed between prototypes 4 and 5 (*p* = 0.0072). The release kinetics are shown in the lower right section of Figure 2b. The results reveal that both emulsifier-containing prototypes released L-Lys more rapidly than prototype 1. At 8 h, prototypes 4 and 5 reached 92.7% and 90.0% release, respectively, significantly exceeding that of prototype 1 (73.9%; *p* < 0.0001 vs. prototype 4; *p* = 0.0037 vs. prototype 5). No significant difference was observed between prototypes 4 and 5 at 8 h (*p* = 0.4024). The release kinetics are shown in Figure 2. The two-way ANOVA results show that time accounted for 95.87% of the total variation (*p* < 0.0001), prototype for 1.85% (*p* < 0.0001), and the time × prototype interaction for 1.47% (*p* < 0.0001), indicating that both factors and their interaction significantly influenced L-Lys release.

### 3.4. In Vivo Post-Prandial Plasma Kinetics Evaluation

**Figure 3:**
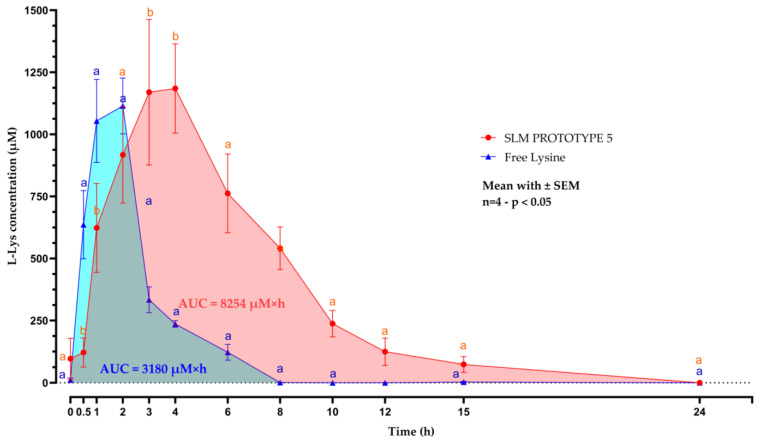
Plasma concentration–time (µM/h) profile of L-lysine (L-Lys) following oral administration of free L-Lys (blue line plot) or solid lipid microcapsules (SLM prototype 5; red line plot) in weaned pigs. Blood samples were collected at defined intervals over 24 h, and L-Lys concentrations were measured using LC–MS/MS. Data are presented as means ± SEM (n = 4). A two-way ANOVA followed by Šidák’s post hoc analysis was performed to evaluate the interaction between the two independent variables: prototype and time. Different letters (a, b) indicate statistically significant differences at each time point (*p* < 0.05). AUC = area under the curve.

Key post-prandial plasma kinetics parameters are summarized in Table 3, and the plasma concentration–time curve is shown in Figure 3. Following oral administration, prototype 5 SLMs demonstrated a delayed and sustained release of L-Lys compared with the free form. The encapsulated L-Lys group reached the peak plasma concentration (C. max.) of 1185 µM at 3–4 h post-administration, significantly later than the free L-Lys group (with a C. max. of 1115 µM at 1 h). The area under the curve (AUC_0–24h_) was significantly greater for the SLMs (8254 µM × h) compared with the free L-Lys (3180 µM × h; *p* < 0.0001), indicating enhanced bioavailability.

Furthermore, plasma L-Lys levels remained detectable for up to 24 h in the SLM group, whereas concentrations in the free L-Lys group dropped below detectable levels within 8 h. L-Lys SLMs maintained significantly higher plasma levels between 0.5 h and 6 h (*p* between 0.0001 and 0.0479). The two-way ANOVA results show that time had a statistically significant effect (*p* < 0.0001), while prototype was not significant (*p* = 0.1205). However, the time × prototype interaction was highly significant (*p* < 0.0001), indicating that the effect of time on L-Lys release depended on the prototype used.

## 4. Discussion

This study evaluated the impact of the matrix formulation on optimizing L-Lys delivery for swine nutrition. Specifically, it showed how lipid compositions and the emulsifier concentration could influence both the in vitro gastric retention and intestinal release dynamics of L-Lys SLMs, obtained via the spray-chilling technique.

This method offers a more suitable alternative to other existing approaches. For example, spray drying typically requires elevated temperatures [19], which can compromise thermolabile compounds such as L-Lys. Considering that L-Lys degradation starts at approximately 100–120 °C and accelerates above 150 °C [8], processing this compound at lower temperatures is essential. Conversely, coacervation often involves the use of organic solvents, which are not usually safe and can lead to the denaturation or degradation of delicate biomolecules like L-Lys [20]. In contrast, spray chilling not only works at low temperatures and does not require the use of organic solvents but also provides excellent reproducibility. In this work, the resulting EE of 97.81–102.33% with minimal variability (SD ≤ 1.26) confirmed the method’s robustness.

Building upon existing knowledge, a related study on SLMs explored the influence of physical characteristics, such as particle size and β-crystallinity, on lipid matrices composed of palm oil and 1% rapeseed lecithin. Their findings revealed that these factors can influence the release of active agents (such as AAs) in a simulated gastrointestinal environment [21]. The present study extended that work by optimizing the lipid matrix composition and investigating its influence on SLMs’ performance [8,22]. Our findings indicate that SLMs composed of saturated C16:0-rich TGs and monounsaturated C18:1 TGs provided superior gastric retention and controlled release over 8 h, significantly outperforming those based on free fatty acids. These observations are consistent with the broader literature on encapsulation, which reports that long-chain saturated TGs form semi-crystalline matrices, enhancing structural rigidity and resistance to acid-induced degradation [22,23].

Moreover, this study demonstrated that SLMs based on monounsaturated C18:1 TGs (prototype 2) exhibited slower intestinal breakdown, potentially delaying nutrient release beyond the proximal absorption sites, where L-Lys uptake is most efficient [24]. This aligns with Bergen et al. (2023), who reported altered intestinal cell dynamics with varying fatty acid profiles [25]. The contrasting rates of intestinal release observed between prototypes 1 (rapid) and 2 (slow), despite their comparable gastric retention, strongly suggest that even minor alterations in the lipid composition (the saturated/unsaturated fatty acid ratio or the resulting crystal structure) significantly influence the active compound release in the intestinal environment. As reported in the literature, intestinal release is closely tied to lipid hydrolysis, a process governed by the surface area available for enzymatic action [21]. Additionally, the crystalline structure and surface morphology of the lipid particles play a crucial role by potentially hindering the diffusion of water and encapsulated compounds (such as L-Lys) through the matrix’s pores [21]. A more ordered and less porous surface, along with a more stable crystalline structure (such as the β-phase), tends to slow down release [21]. It is plausible that the specific TG profiles of prototypes 1 and 2 resulted in distinct crystalline structures or surface morphologies, explaining their different intestinal release kinetics, even though both provided excellent gastric retention.

Furthermore, the results of this study demonstrate significantly reduced gastric retention and rapid, nearly complete, intestinal release for the free fatty acid-based formulation (prototype 3), contrasting with the performance of TG-based prototypes. While previous studies reported that long-chain saturated fatty acids (e.g., stearic and arachidic acids) can form SLMs resistant to in vitro gastric and intestinal digestion [22,26], our findings suggest that a matrix primarily composed of free fatty acids, under the specific system and encapsulation method used in this study, may not offer equivalent protection against acidic gastric conditions or prevent rapid dispersion/dissolution. This difference may arise from variations in how free fatty acids are organized within the matrix compared with TGs. In addition, it can be explained also by the enhanced tendency of free fatty acids to form micelles or other structures in the digestive environment, promoting faster release. These findings highlight the importance of the raw material selection for optimal encapsulation and release performance, a principle previously studied in ruminant nutrition using wax-based matrices (e.g., carnauba or beeswax) for methionine and lysine protection [27,28].

This study also demonstrated the modulatory effects of emulsifier inclusion on SLMs’ performance. The addition of 1% emulsifier significantly enhanced intestinal L-Lys release (~90% over 8 h) compared with the formulation lacking emulsifier. Conversely, a 0.5% emulsifier content provided significantly lower gastric retention (87%). This dose-dependent effect underscores the importance of the emulsifier concentration in modulating matrix permeability. Emulsifiers likely accelerate intestinal release by improving the wettability of the lipid matrix in aqueous intestinal environments, facilitating water penetration into particles and enabling faster dissolution and diffusion of encapsulated water-soluble AAs, like L-Lys. Moreover, emulsifiers can interact with intestinal bile salts, promoting gradual disintegration and sustained nutrient delivery [22].

The greater percentage of L-Lys release from the 0.5% emulsifier formulation under simulated gastrointestinal conditions compared with the 1% formulation highlights the substantial difference in the early release kinetics, with implications for metabolic optimization. The accelerated release from the 0.5% formulation can increase AA availability in the upper gastrointestinal tract (stomach and duodenum/proximal jejunum) [8,21,26]. This premature release can result in rapid, unbalanced absorption that may not align with immediate metabolic requirements and the absorption patterns of other AAs, thereby increasing L-Lys oxidation and catabolism [6]. Conversely, the 1% emulsifier formulation exhibits slower release in the initial phases and can promote a potentially prolonged availability of L-Lys along the intestinal tract [8], reaching the typical objectives of controlled-release formulations [6,26].

In vivo, the shift in the L-Lys plasma peak from 1 h (free form) to 3–4 h (L-Lys SLMs) indicates slower absorption, as suggested also by previous works [5,6]. The detectable plasma levels up to 24 h for L-Lys SLMs (compared with less than 8 h for the free form) confirm prolonged release. This slow release and higher sustained absorption can lead to a stable plasma concentration over time. The area under the curve for the L-Lys SLMs was 2.6-fold higher than the one for free Lys (8254 vs. 3180 µM × h), indicating that a greater quantity of L-Lys reached the systemic circulation over the 24 h period. These results are consistent with previous in vivo studies in pigs and poultry, supporting the concept that slow release may prevent intestinal transporter saturation, improve absorption efficiency, and reduce nitrogen excretion [29,30]. Specifically, intestinal L-Lys uptake follows Michaelis–Menten kinetics (KM ≈ 0.2 mM) [31]. Bröer et al. (2023) reported that a jejunal L-Lys concentration of 0.6–6 mM, measured after an ordinary meal, is already sufficient to saturate the apical transporter in swine [3]. A practical supplement of 0.25% crystalline L-Lys raised luminal L-Lys to ≈ 7 mM, thereby extending the saturated period. The resulting early portal spike (0.5 h vs. 2.5 h) can be flattened by micro- or nano-encapsulated L-Lys [4], which lowers urinary nitrogen without compromising growth [5]. A study on broilers by Sun et al. (2020) [6] indicated that encapsulated AAs permitted a 20% reduction in supplementation levels without impairing productive performance, suggesting a greater AA absorption capacity or utilization efficiency compared with the crystalline form. These supplements modulated plasma AAs’ profiles by avoiding post-prandial spikes and enhancing gut morphology and transporter expression [6]. This further supports the idea of improved bioavailability and utilization with the encapsulated form. Similar improvements in AAs’ utilization via encapsulation have been shown in sheep, where bypass formulations of lysine and methionine using hydrogenated fats or waxes enhanced milk yield, wool quality, and carcass traits [28]. Although promising, these systems remain largely experimental and lack the industrial feasibility of spray-chilled SLMs.

By providing a sustained L-Lys release profile that aligns with physiological demand, the SLMs developed in this study can contribute to more precise AA nutrition, allowing reduced crude protein formulations and mitigating nitrogen losses. This parallels the sustainability outcomes observed in pigs and poultry fed encapsulated AAs [5,6] and supports current efforts in circular agriculture and One Health strategies [32]. Moreover, the use of GRAS-approved hydrogenated vegetable oils and emulsifiers in the encapsulation matrix ensures regulatory compliance and facilitates immediate adoption in commercial feed manufacturing, unlike nanoparticle systems, which face hurdles in cost, safety assessment, and legislative frameworks [33,34] While this study confirms the efficacy of optimized SLMs in enhancing L-Lys’s relative bioavailability in swine, several limitations should be considered. First, the in vitro digestion model, although useful for preliminary screening, does not fully reflect the complex dynamics of the gastrointestinal tract in vivo. Factors such as enzymatic variability and gut transit time are difficult to fully replicate in vitro, while interactions with the gut microbiota are often not achievable in a standard digestion model. Therefore, further in vivo validation under commercial farming conditions is needed to confirm these findings in a more realistic setting. Second, while this study focused on matrix composition and emulsifier concentration, other formulation variables, such as particle size distribution and surface characteristics, may also influence nutrient release kinetics and should be explored in future optimization studies. Additionally, evaluating the matrix behavior at higher emulsifier concentrations and testing SLMs’ long-term stability under industrial feed-processing conditions (e.g., pelleting) would improve their practical applicability.

Further studies using other animal models could offer broader insights into the versatility and adaptability of the SLMs’ formulations across species. Additionally, comparative studies involving alternative encapsulation systems, such as biopolymer-based matrices (e.g., alginate and pectin) or hybrid systems combining emulsion-based delivery with solid microcapsules, could help contextualize the advantages of solid lipid matrices in terms of protection, scalability, and cost-effectiveness.

## 5. Conclusions

This study demonstrated that the composition of lipid matrices and the inclusion of emulsifiers are critical parameters in optimizing the performance of SLMs for L-Lys delivery in swine. The lipid composition of the encapsulating matrix has a key role in determining the extent of gastric retention and the kinetics of intestinal release. Triglycerides offer higher retention than free fatty acids, and even minor variations within triglyceride-based matrices can influence release. Additives such as emulsifiers can affect the matrix properties and modulate release, potentially increasing it in the intestinal phase at higher concentrations. Formulations based on saturated C16:0-rich TGs combined with 1% emulsifier provided superior gastric retention and enhanced intestinal release in vitro. In the in vivo model, encapsulation modulated the post-prandial plasma kinetics, resulting in a delayed and prolonged release of L-Lys and, crucially, a higher overall relative bioavailability compared with the free form. These findings suggest that a carefully designed lipid matrix for encapsulation is essential to optimize both the protection of the AA in the stomach and its controlled release in the intestine, thereby enhancing its absorption and utilization efficiency by the animal. By ensuring sustained nutrient availability and reducing premature losses, this approach can contribute to improved feed efficiency, reduced nitrogen excretion, and greater alignment with sustainable swine nutrition.

## Figures and Tables

**Table 1:** Compositions of solid lipid microcapsule (SLM) prototypes.

Prototype	Lipid Matrix Composition	Emulsifier % (*w*/*w*)
1	Hyd. vegetable oil rich in saturated C16:0-TGs	-
2	Hyd. vegetable oil rich in monounsaturated C18:1-TGs	-
3	Saturated free fatty acid preparation (≥95% C18:0)	-
4	Hyd. vegetable oil rich in saturated C16:0-TGs	0.5%
5	Hyd. vegetable oil rich in saturated C16:0-TGs	1%

Hyd. = hydrogenated; TGs = triglycerides.

**Table 2:** Prototypes’ compositions.

Prototype	Encapsulation Efficiency (%) ± SD *
1	102.33 ± 1.26
2	97.81 ± 0.25
3	99.07 ± 0.26
4	100.60 ± 1.12
5	101.78 ± 0.61

* Values expressed as means ± standard deviation (n = 4 technical replicates).

**Table 3:** Post-prandial plasma kinetics parameters of L-lysine (L-Lys) treatments.

Treatment	C. Max. (µM)	T. Max. (h)	AUC_0–24h_ (µM × h)
Free L-Lys	1115	1	3180
L-Lys SLMs ^1^	1185	3–4	8254
Placebo	≤0.1	-	-

^1^ Prototype 5 was used. AUC = area under the curve.

## Data Availability

The data presented in this study will be made available upon request from the corresponding author or other co-authors.

## Supplementary Materials

The following supporting information can be downloaded at https://www.mdpi.com/article/10.3390/ani15121806/s1: Table S1: Key parameters and conditions for chromatographic and mass spectrometric analysis. Figure S1: In vitro retention (%) profiles of L-lysine (L-Lys) from solid lipid microcapsule (SLM) prototypes during simulated gastrointestinal digestion. Figure S2: In vitro retention (%) profiles of L-lysine (L-Lys) from solid lipid microcapsule (SLM) prototypes.

## References

[1] Hasan M.S., Crenshaw M.A., Liao S.F. (2020). Dietary Lysine Affects Amino Acid Metabolism and Growth Performance, Which May Not Involve the GH/IGF-1 Axis, in Young Growing Pigs. J. Anim. Sci..

[2] Batterham E.S. (1985). Amino Acid Availability in Pig Diets with Special Reference to Natural Proteins and Synthetic Amino Acids. Recent Developments in Pig Nutrition.

[3] Bröer S. (2023). Intestinal Amino Acid Transport and Metabolic Health. Annu. Rev. Nutr..

[4] Yen J.T., Kerr B.J., Easter R.A., Parkhurst A.M. (2004). Difference in Rates of Net Portal Absorption between Crystalline and Protein-Bound Lysine and Threonine in Growing Pigs Fed Once Daily. J. Anim. Sci..

[5] Prandini A., Sigolo S., Morlacchini M., Grilli E., Fiorentini L. (2013). Microencapsulated Lysine and Low-Protein Diets: Effects on Performance, Carcass Characteristics and Nitrogen Excretion in Heavy Growing–Finishing Pigs. J. Anim. Sci..

[6] Sun M., Jiao H., Wang X., Uyanga V.A., Zhao J., Lin H. (2020). Encapsulated Crystalline Lysine and DL-Methionine Have Higher Efficiency than the Crystalline Form in Broilers. Poult. Sci..

[7] Contreras-López G., Carrillo-López L.M., Vargas-Bello-Pérez E., García-Galicia I.A. (2024). Microencapsulation of feed additives with potential in livestock and poultry production: A systematic review. Chil. J. Agric. Anim. Sci..

[8] Nahum V., Domb A.J. (2021). Recent Developments in Solid Lipid Microparticles for Food Ingredients Delivery. Foods.

[9] Ibrahim S.L., Hassen A. (2021). Characterization, Density and In Vitro Controlled Release Properties of Mimosa (*Acacia mearnsii*) Tannin Encapsulated in Palm and Sunflower Oils. Animals.

[10] Atkins P.W., de Paula J. (2010). Atkins’ Physical Chemistry.

[11] Pande G., Akoh C.C., Lai O.-M. (2012). Food Uses of Palm Oil and Its Components. Palm Oil.

[12] Rondou K., Romanus M., Verwee E., Penagos I.A., De Witte F., Skirtach A.G., Dewettinck K., Van Bockstaele F. (2024). Crystallization Behavior and Structural Build-Up of Palm Stearin—Wax Hybrid Fat Blends. Food Biophys..

[13] Miskandar M.S., Che Man Y.B., Abdul Rahman R., Nor Aini I., Yusoff M.S.A. (2006). Effects of Emulsifiers on Crystallization Properties of Low-melting Blends of Palm Oil and Olein. J. Food Lipids.

[14] Johansson D., Bergenståhl B. (1995). Lecithins in Oil-continuous Emulsions. Fat Crystal Wetting and Interfacial Tension. J. Am. Oil Chem. Soc..

[15] Brodkorb A., Egger L., Alminger M., Alvito P., Assunção R., Ballance S., Bohn T., Bourlieu-Lacanal C., Boutrou R., Carrière F. (2019). INFOGEST Static in Vitro Simulation of Gastrointestinal Food Digestion. Nat. Protoc..

[16] Henze L.J., Koehl N.J., Bennett-Lenane H., Holm R., Grimm M., Schneider F., Weitschies W., Koziolek M., Griffin B.T. (2021). Characterization of Gastrointestinal Transit and Luminal Conditions in Pigs Using a Telemetric Motility Capsule. Eur. J. Pharm. Sci..

[17] Wang H., Pampati N., McCormick W.M., Bhattacharyya L. (2016). Protein Nitrogen Determination by Kjeldahl Digestion and Ion Chromatography. J. Pharm. Sci..

[18] AccQ-Tag Ultra Derivatization Kit. https://www.waters.com/nextgen/us/en/shop/application-kits/186003836-accq-tag-ultra-derivatization-kit.html.

[19] Tao Y., Tang Z., Huang Q., Xu X., Cheng X., Zhang G., Jing X., Li X., Liang J., Granato D. (2024). Effects of Spray Drying Temperature on Physicochemical Properties of Grapeseed Oil Microcapsules and the Encapsulation Efficiency of Pterostilbene. LWT.

[20] Dixit K., Athawale R.B., Singh S. (2015). Quality Control of Residual Solvent Content in Polymeric Microparticles. J. Microencapsul..

[21] Rajendrakumar S., Beaumal V., Kermarrec A., Lopez C., Novales B., Rabesona H., Simongiovanni A., Demersay T.C., Marze S. (2024). Release Profile of Amino Acids Encapsulated in Solid Lipid Particles during in Vitro Oro-Gastrointestinal Digestion. Food Res. Int..

[22] Rosiaux Y., Jannin V., Hughes S., Marchaud D. (2014). Solid Lipid Excipients—Matrix Agents for Sustained Drug Delivery. J. Control. Release.

[23] De Witte F., Penagos I.A., Rondou K., Moens K., Lewille B., Tzompa-Sosa D.A., Van De Walle D., Van Bockstaele F., Skirtach A.G., Dewettinck K. (2024). Insights in the Structural Hierarchy of Statically Crystallized Palm Oil. Crystals.

[24] Liao S.F., Wang T., Regmi N. (2015). Lysine Nutrition in Swine and the Related Monogastric Animals: Muscle Protein Biosynthesis and Beyond. SpringerPlus.

[25] Bergen J., Karasova M., Bileck A., Pignitter M., Marko D., Gerner C., Del Favero G. (2023). Exposure to Dietary Fatty Acids Oleic and Palmitic Acid Alters Structure and Mechanotransduction of Intestinal Cells in Vitro. Arch. Toxicol..

[26] Albuquerque J., Neves A.R., Van Dorpe I., Fonseca A.J.M., Cabrita A.R.J., Reis S. (2023). Production of Rumen- and Gastrointestinal-Resistant Nanoparticles to Deliver Lysine to Dairy Cows. Sci. Rep..

[27] Carvalho Neto J.P.D., Bezerra L.R., Da Silva A.L., De Moura J.F.P., Pereira Filho J.M., Da Silva Filho E.C., Guedes A.F., Araújo M.J., Edvan R.L., Oliveira R.L. (2019). Methionine Microencapsulated with a Carnauba (*Copernicia prunifera*) Wax Matrix for Protection from Degradation in the Rumen. Livest. Sci..

[28] Inô C.F.A., Pereira Filho J.M., De Oliveira R.M.T., De Oliveira J.F.P., Da Silva Filho E.C., Nascimento A.M.D.S.S., Oliveira R.L., Do Nascimento R.R., De Lucena K.H.D.O.S., Bezerra L.R. (2024). New Technology of Rumen-Protected Bypass Lysine Encapsulated in Lipid Matrix of Beeswax and Carnauba Wax and Natural Tannin Blended for Ruminant Diets. Animals.

[29] Roy N., Lapierre H., Bernier J.F. (2000). Whole-Body Protein Metabolism and Plasma Profiles of Amino Acids and Hormones in Growing Barrows Fed Diets Adequate or Deficient in Lysine. Can. J. Anim. Sci..

[30] Le P.D., Aarnink A.J.A., Jongbloed A.W. (2009). Odour and Ammonia Emission from Pig Manure as Affected by Dietary Crude Protein Level. Livest. Sci..

[31] Woodward A.D., Fan M.Z., Geor R.J., McCutcheon L.J., Taylor N.P., Trottier N.L. (2012). Characterization of L-Lysine Transport across Equine and Porcine Jejunal and Colonic Brush Border Membrane. J. Anim. Sci..

[32] Shurson G.C., Urriola P.E. (2022). Sustainable Swine Feeding Programs Require the Convergence of Multiple Dimensions of Circular Agriculture and Food Systems with One Health. Anim. Front..

[33] Ashfaq R., Rasul A., Asghar S., Kovács A., Berkó S., Budai-Szűcs M. (2023). Lipid Nanoparticles: An Effective Tool to Improve the Bioavailability of Nutraceuticals. Int. J. Mol. Sci..

[34] Singh M.N., Hemant K.S.Y., Ram M., Shivakumar H.G. (2010). Microencapsulation: A Promising Technique for Controlled Drug Delivery. Res. Pharm. Sci..

