# Supplementary material for: Optimization of Solid Lipid Microcapsule Matrix for Enhanced Release and Bioavailability of L-Lysine in Swine
Source: Animals (Basel). 2025 Jun 19;15(12):1806. doi: 10.3390/ani15121806 (PMC12189550; doi:10.3390/ani15121806)

Supplementary Materials:

Table S1. Key parameters and conditions for chromatographic and mass spectrometric analysis.

**Chromatographic Parameters**	**Conditions**		
**Mobile Phase**	Solvent A: H_2O (LC-MS grade), 0.1% formic acid ≥ (99%)<br>Solvent B: ACN (LC-MS grade), with 0.1% formic acid ≥ (99%)		
**Gradient Profile**	**Time (min)**	**A(%)**	**B(%)**
	0.0	99	1
	1.0	99	1
	2.0	87	13
	5.5	85	15
	6.5	5	95
	7.5	5	95
	7.6	99	1
	9.0	99	1
**Flow Rate**	0.5 mL/min		
**Injection Volume**	5 µL		
**Column**	Waters CORTECS™ Premier C18 (1.6 µm, 2.1 × 150 mm)		
**Column Oven Temperature**	55 °C		
**Autosampler Temperature**	10 °C		
**Run Time**	9 min		

**Mass Spectrometry Parameters**	**Conditions**
**Ionization Mode**	Multiple Reaction Monitoring (MRM) in ESI+.
**Analyte**	L-Lys+AccQTag (244.10 - 171.100 m/z)
**Cone Voltage**	20 V
**Collision Energy**	30 V
**General MS Settings**	
**Capillary Voltage**	1 kV
**Desolvatation Temperature**	600°C
**Desolvatation Gas Flow**	1000 L/h
**Cone Gas Flow**	100 L/h
**Source Temperature**	150 °C

Figure S1. In vitro retention (%) profiles of L-lysine (L-Lys) from solid lipid microcapsule (SLMs) prototypes formulated with emulsifier during simulated gastrointestinal digestion.

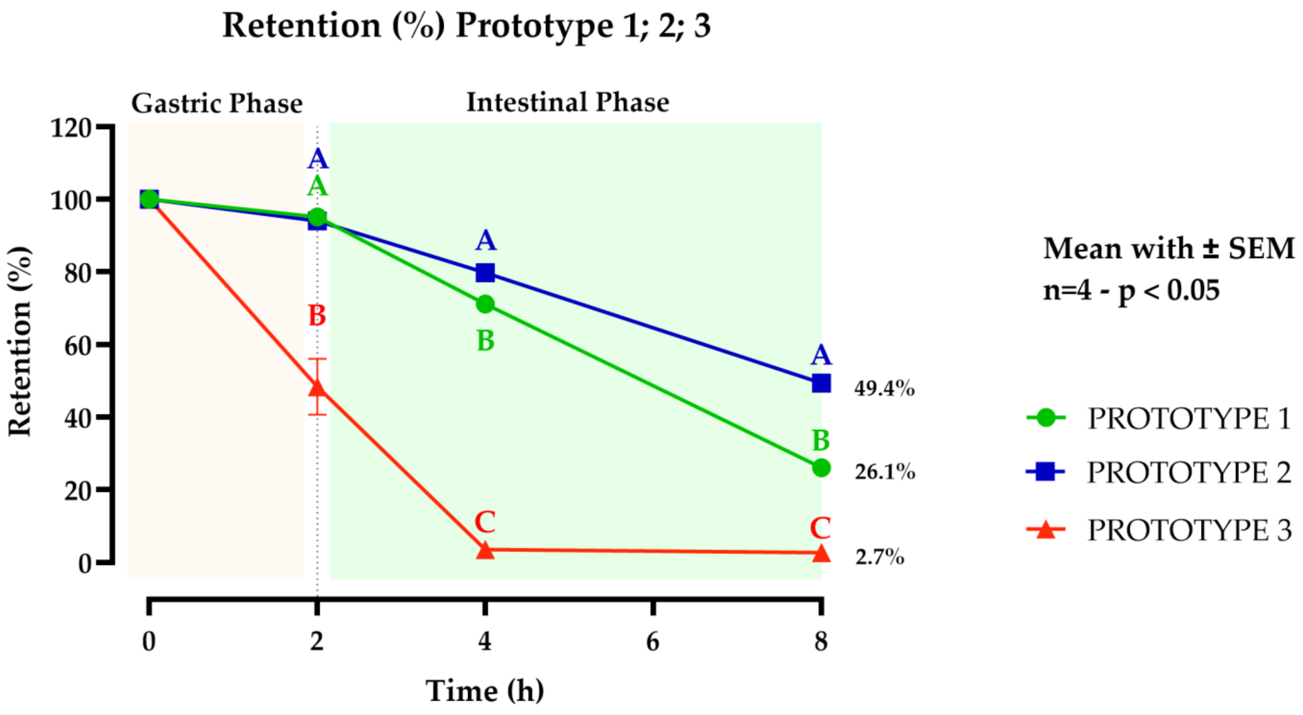

Figure S2. In vitro retention (%) profiles of L-lysine (L-Lys) from solid lipid microcapsule (SLMs) prototypes during simulated gastrointestinal digestion.

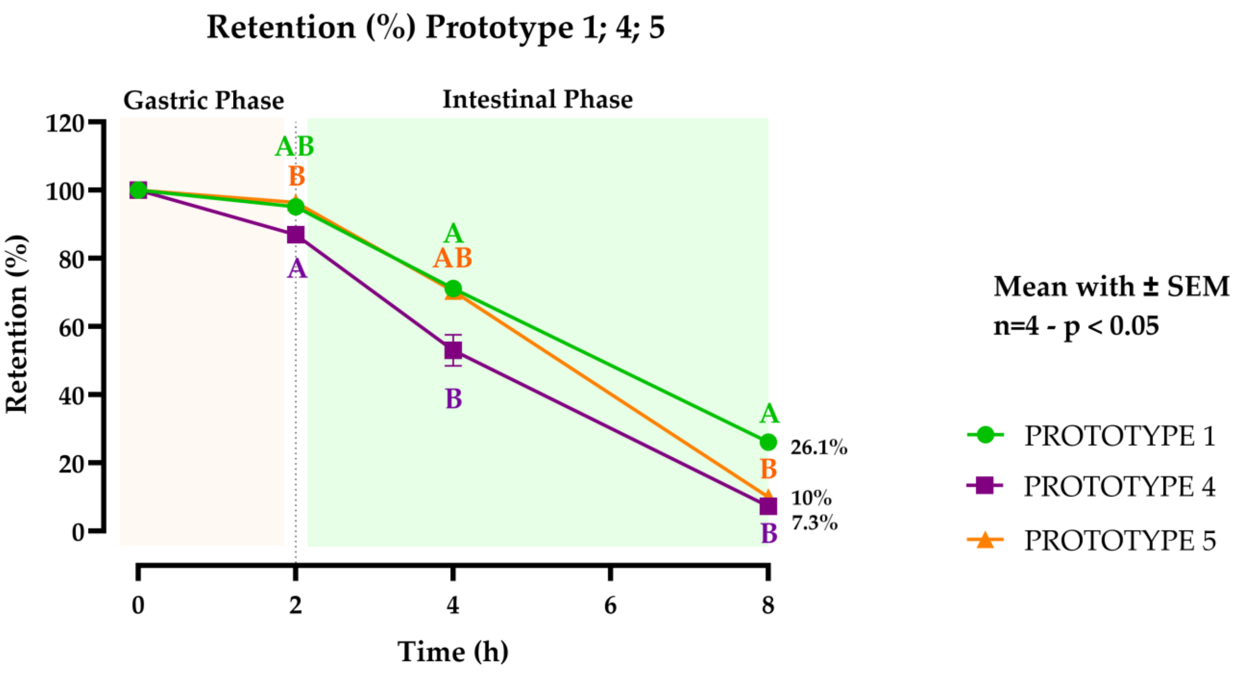

Supplement: Supplementary file 1 [file animals-15-01806-s001.zip › animals-3669281-supplementary.pdf]
